# A Review of Enzymatic Transesterification of Microalgal Oil-Based Biodiesel Using Supercritical Technology

**DOI:** 10.4061/2011/468292

**Published:** 2011-09-11

**Authors:** Hanifa Taher, Sulaiman Al-Zuhair, Ali H. Al-Marzouqi, Yousef Haik, Mohammed M. Farid

**Affiliations:** ^1^Chemical and Petroleum Engineering Department, UAE University, Al-Ain 17555, United Arab Emirates; ^2^Mechanical Engineering Department, UAE University, Al-Ain 17555, United Arab Emirates; ^3^Chemical and Materials Engineering Department, University of Auckland, 1142 Auckland, New Zealand

## Abstract

Biodiesel is considered a promising replacement to petroleum-derived diesel. Using oils extracted from agricultural crops competes with their use as food and cannot realistically satisfy the global demand of diesel-fuel requirements. On the other hand, microalgae, which have a much higher oil yield per hectare, compared to oil crops, appear to be a source that has the potential to completely replace fossil diesel. Microalgae oil extraction is a major step in the overall biodiesel production process. Recently, supercritical carbon dioxide (SC-CO_2_) has been proposed to replace conventional solvent extraction techniques because it is nontoxic, nonhazardous, chemically stable, and inexpensive. It uses environmentally acceptable solvent, which can easily be separated from the products. In addition, the use of SC-CO_2_ as a reaction media has also been proposed to eliminate the inhibition limitations that encounter biodiesel production reaction using immobilized enzyme as a catalyst. Furthermore, using SC-CO_2_ allows easy separation of the product. In this paper, conventional biodiesel production with first generation feedstock, using chemical catalysts and solvent-extraction, is compared to new technologies with an emphasis on using microalgae, immobilized lipase, and SC-CO_2_ as an extraction solvent and reaction media.

## 1. Introduction

Continuous exploration and consumption of fossil fuels have led to a decline in worldwide oil reserves. As the world energy demand is continuously increasing, the most sufficient way to meet the growing demand is by finding alternative fuels. From the point of environment protection, finding alternative fuels that are sustainable and environment friendly is essential. 

More than a century ago, Rudolf Diesel tested the suitability of using vegetable oils as fuel in his engine [1, 2]. In the 1930s and 1940s, vegetable oils were used as a diesel fuel for emergency situations. At that time, vegetable oil fuels were not competitive because they were more expensive than petroleum fuels, and therefore the idea was abandoned. With the worries about petroleum fuel availability and latest increases in petroleum prices, using vegetable oils in diesel engines has regained attention. 

A number of studies have shown that triglycerides (TGs) hold promise as alternative diesel engine fuels [2, 3]. This has an advantage of being available, renewable with higher cetane number, and biodegradable [4–6]. However, the main disadvantage of oils is their high viscosity and low volatility [2, 7, 8]. Therefore, direct use of TGs is generally unacceptable and not practical since it causes engine coking, carbon depositing and gelling of the lubricating oil [8–10]. To overcome these problems, dilution, pyrolysis, cracking, and transesterification of the oil have been suggested [5, 11]. Among all these methods, transesterification has been used widely as a favorable method. Transesterification reaction of TGs, known as alcoholysis, is an important reaction that produces fatty acids alkyl esters (FAAE) [8, 12]. It was reported that replacing petroleum diesel with FAAE results in a reduction of unburned hydrocarbons, carbon monoxide (CO), and particular matter (PM) formation [13, 14]. Several methods of transesterification using alkali catalysts [9, 14–18], acid catalysts [17, 19–23], and enzyme lipase in presence and absence of solvents have been reported [24–29]. Most of the commercial biodiesel processes require the use of a catalyst, which requires a recovery unit to separate reaction products and remove the catalyst. These disadvantages of using catalyst could be eliminated by carrying out noncatalytic reaction. Sake and Kusdiana [30] developed a method using supercritical methanol (SCM) where triglycerides fatty acids were converted to methyl esters without using any catalyst. Sake and Kusdiana [30] and Madras et al. [31] reported the advantage of using supercritical alcohols (SCA), especially methanol, whereas a process requires short reaction time and no need for reaction product separation from the solvent. However, this process is energy intensive as it is carried out at the supercritical conditions of methanol. Nevertheless, based on van Kasteren and Nisworo [32] economic assessment, this process appears to be feasible.

Enzymatic biodiesel approach showed promising results due to their high selectivity and mild operative conditions. Enzymatic transesterification reaction is similar to conventional transesterification, except that they are catalyzed by a variety of biological catalysts rather than chemical catalysts. In contrast to conventional processes, biocatalysts can transesterify TGs with a high free fatty acid (FFA) content [33]. Lipase-catalyzed transesterification of TG has been investigated by several investigators [33–37]. One common drawback with the use of enzyme-based processes is the high cost of the enzyme compared to conventional chemical catalysts; therefore, their recycle is required, which is possible through enzyme immobilization. 

Immobilization of enzymes has generally been used to attain reusable enzyme with lower production cost [25, 38, 39]. Thus, immobilized form of lipase has been used in most of transesterification processes [25, 36, 40]. Besides enzyme reusability, other advantages of using immobilized lipase as a catalyst are enhanced activity and stability [41, 42]. 

Several researches have been carried out to produce biodiesel in solvent systems. Presently, industries are facing problems in using conventional solvents due to environmental worries. In the last couple of decades, enzyme-catalyzed reactions in supercritical carbon dioxide (SC-CO_2_) has been studied. Previously, most of the studies were investigating the feasibility of using biocatalyst in SC-CO_2_, whereas recent studies are focusing on obtaining good yield and conversions. 

Vegetable oils consist of TG of straight chains of fatty acids. With the high cost of biodiesel produced from vegetable oils, researchers are looking for low-cost feedstocks. For that waste oils, cooking oils and fats from animal sources were proposed. The main drawback of using animal fats is their high melting points, which may require the use of organic solvents. However, organic solvent use requires a solvent recovery unit and energy needed for its separation. To overcome this, supercritical fluids (SCFs) were introduced.

During the past decades, SCFs have been investigated as alternative solvents for reactions rather than using conventional solvents. Among all supercritical fluids, SC-CO_2_ is the most appropriate choice as a consequence of its availability. In general, CO_2_ is nontoxic, nonflammable, environmentally friendly, and recyclable fluid [43]. Thus, reactions in SC-CO_2 _ media become the preferable route for chemical synthesis.

Conventionally, biodiesel is produced from vegetable oils, animal fats, and waste cooking oils [7, 30, 31, 44–46]. However, these feedstocks are inefficient and unsustainable [47]. Furthermore, using vegetable oil as a fuel source competes with its use as food and proposes for land development in order not to compete with food and land. On the other hand, animal fat cannot be considered as a continuous supply of feed stock [48]. Thus, biodiesel production using these feedstocks, realistically, cannot replace all world biodiesel requirements. 

In contrast, microalgae have been recognized as a promising alternative source for biodiesel production. They are a group of organisms that can grow photosynthetically and accumulate large amounts of lipids [49, 50]. According to Sheehan et al. [50], if microalgal oil production could be scaled up, less than 6 million hectares would be required to meet current fuel demands.

Considering the above facts, this paper provides an overview on biodiesel production from microalgae with a particular emphasis on the use of microalgae as a promise feedstock, lipase as promise catalyst, and SC-CO_2_ as a promise extraction solvent and reaction media.

## 2. Biodiesel

Biodiesel has arisen as a possible alternative for petroleum diesel because of the similarities that biodiesel has with petroleum diesel [39, 51]. Biodiesel fuel has many advantages over petroleum fuel such as being nontoxic, biodegradable, renewable, and do not contribute to net accumulation of the green house gases [52, 53]. Also, biodiesel has lower sulfur and aromatic content, higher cetane number, and flash point than petroleum diesel [5, 7, 54–56]. Other benefits of biodiesel include increased lubricity and lower emissions of certain harmful exhaust gases in comparison to petroleum diesel fuel [55]. 

Comparing petroleum diesel fuel to biodiesel, Schumacher et al. [14] reported that biodiesel results in a 45% reduction in total hydrocarbon emissions, 47% reduction in CO emissions, and 66% reduction in PM emissions, whereas, Demirbas [55] reported a 42% reduction in CO and 55% in PM emissions relative to standard diesel fuel. These effects are generally attributed to the higher cetane number and oxygen content of biodiesel fuel. Although the biodiesel environmental considerations are very positive, biodiesel increases nitrogen oxides (NO_x_) emissions. However, reports show that reductions in NO_x_ emissions are possible with some modifications in combustion temperatures and injection timing [57, 58].

As mentioned earlier, direct use of vegetable oil has several negative aspects, such as their high viscosity and low volatility, which lead to incomplete combustion in diesel engines, therefore, carbon deposition [5, 9, 59, 60]. However, the direct use of vegetable oils as biodiesel may be possible by mixing them with conventional diesel in an appropriate ratio, but this mixing will be impractical for long-term uses in the engine due to the high viscosity, low stability, acid composition, and FFA content [4, 61, 62]. Therefore, considerable efforts have been made to develop vegetable oil derivatives that have properties near those of the petroleum-based diesel fuels.

Pyrolysis (cracking), microemulsion, and transesterification are the possible methods to minimize problems associated with feedstock use [5, 8, 11]. The first two methods are costly and yield low quality biodiesel, whereas the latter, transesterification, is the most common method to transform oil into biodiesel, which is the focus of this paper.

### 2.1. Transesterification

Transesterification is the common method used to transform TG into biodiesel. This consists of the reaction between TG and an acyl-acceptor [11, 63]. Carboxylic acids, alcohols, or another ester can be used as acyl-acceptor. Transesterification produces glycerol when alcohol is used as acyl-acceptor or triacylglycerol when ester is used [8, 56, 60, 62, 64]. Transesterification process using a catalyst is called catalytic transesterification process, whereas that without catalyst is called noncatalytic transesterification process [8, 10, 65, 66]. Moreover, catalytic process is divided into two types: homogenous and heterogeneous processes depending on the catalyst used. 

Transesterification is a chemical process of transforming large and branched TG into smaller and straight chain molecules, which is similar in size to the molecules of the species present in diesel fuel [67, 68]. Stoichiometrically, for each mole of TG three moles of alcohol are required. But in general, a higher molar ratio of alcohol is used in order to achieve maximum biodiesel production. This molar ratio depends on the type of used feedstock, type of catalyst, temperature, and reaction time. Methanol, ethanol, and propanol are the most commonly used alcohols. In fact, biodiesel yield is independent of the type of the alcohol used and the alcohol selection depends on cost [60]. In transesterification, ester bonds are broken first then followed by hydroxyl bond, whereas in esterification hydroxyl bonds are broken before ester bonds resulting in glycerol as byproduct in transesterification and water in esterification [54].

Transesterification can be carried in a number of ways, using different catalytic processes. For example, it can be carried out using alcohol and alkali catalyst, acid catalyst, and biocatalyst or using alcohols in their supercritical state [39, 69]. Overall, transesterification is a sequence of three reactions; TG is first converted to a diacylglycerol (DG) and one fatty acid ester, then the DG is converted to monoacylglycerol (MG) giving an additional fatty acid ester, and finally the MG is converted to glycerol giving the last fatty acid ester. 

Catalyst promotes hydrolysis of the TGs to produce fatty acids and glycerol, with the last being a byproduct. By the end of the transesterification, produced biodiesel and glycerol have to be purified in order to remove the catalyst, which requires a separation step by washing with distilled water for several times. It is well understood that catalyst selection is an important criterion.

#### 2.1.1. Chemical Catalytic Transesterification

**Alkaline Catalyst Transesterification**

A base catalyst is a chemical with a pH value greater than 7. It has the ability to give extra electrons. Sodium hydroxide (NaOH), potassium hydroxide (KOH), and sodium methoxide (CH_3_ONa) are the most common homogeneous base catalysts employed during alkaline transesterification [11, 51, 60]. The base catalyzed process is the most commonly used because of its relative ease. It can be performed at low temperature and pressure and yields high conversion (98%) within a short time [5].

Most important limitation of the base catalysis method is the process sensitivity to both FFA and water contents. It works perfectly when the FFA and moisture contents are less than certain limits, usually below 0.5 wt% for FFA [70, 71]. In case of TGs where FFA contents exceed this limit, pre-treatment step is required. The presence of FFA promotes soap formation, which consumes the catalyst, lowers the yield, and more importantly results in difficult downstream byproducts separation and product purification [7, 8]. About 60–90% of biodiesel cost comes from the high cost of the raw material [7]. In addition, alkali catalyst needs effluent treatment.

Most of the base catalyzed reactions were carried out at temperatures close to the alcohol boiling point with alcohol to oil molar ratio of 6 : 1. Akoh et al. [9] stated that to increase biodiesel yield, a stoichiometric excess of substrates (6 : 1 molar ratio of methanol to oil) is favored.

Homogeneous catalysts have been used industrially for biodiesel production where produced biodiesel and glycerol have to be purified to remove the catalyst. This purification process requires large quantities of water and energy. Thus, heterogeneous catalysts have been suggested to overcome this drawback. Heterogeneous catalysts can be separated easily from the system at the end by filtration and could be reused [60, 72]. Alkaline earth oxides [73], zeolites [3], calcined hydrotalcites [18, 74], and Magnesium and Calcium oxides [16, 72] have been suggested as heterogeneous catalysts and showed good results. However, the high cost of the purified feedstock remains the main problem facing the alkaline catalyzed process.

**Acid Catalyst Transesterification**

The reaction of TGs and alcohol may also be catalyzed with an acid instead of a base. Most commonly used acids are strong acids like sulphuric, sulphonic, phosphoric, and hydrochloric acids [5, 8, 10].

Acid-catalyzed transesterification processes are not as popular as the base-catalyzed processes, mainly because strong acids are corrosive and the processes are too slow. Several reactions may be required in order to achieve high conversion. It has been stated that acid-catalyzed reaction may be 4000 times slower than the base catalyst process [7, 9, 54, 66]. Above that, it requires high amount of alcohol and higher concentration of catalyst. Akoh et al. [9] stated that a molar methanol : oil ratio of 30 : 1 in a range of 55–80°C with 0.5 to 1 mol% catalyst concentration is required to achieve 99% conversion in 50 h. On the other hand, acid-catalyzed processes offer an important advantage for being independent of feedstock FFA content. That is because feedstock FFA is not converted to soap using this kind of catalysts, and hence biodiesel can be produced from low cost feedstock [6, 54].

As mentioned before, feedstock of high FFA content requires a pretreatment step if a base catalyst is to be used. This pretreatment step can be achieved using acid catalysis and methanol, where the FFA is esterified to biodiesel. When equilibrium is reached, the acid catalyst and produced water are removed from the reaction vessel by centrifugation [11]. This is followed by adding fresh methanol and base catalyst to the oil in order to catalyze the transesterification reaction.

Heterogeneous acid catalysts have been also used. This is important to avoid problems associated with homogeneous catalysts. Sulphated tin oxide has been used as superacid catalysts to transesterified waste cooking oil [22]. Sulphated zirconia was also used as catalysts in the alcoholysis of soybean oil and in the esterification of oleic acid [21]. Heteropolyacid was used to transesterify yellow horn oil [75]. Anion and cation exchange resins were used for triolein transesterification reactions with ethanol to produce ethyl oleate [76].

#### 2.1.2. Noncatalytic Transesterification

Although catalysts play a great role in reducing transesterification time, their presence promotes complications of final product purification. This results in increased production process cost.

To avoid catalyst drawbacks, supercritical alcohol (SCA) transesterification process was suggested [13, 51, 77]. SCA transesterification process is a catalyst free process, which provides high conversion of oil to ester in a short time. Tan et al. [78] compared SCM transesterification with conventional catalytic methods. They reported that conventional catalyst required 1 hr to convert palm oil to biodiesel, whereas SCM required only 20 min. As a result of catalyst absence, purification of the products of the transesterification reaction is much simpler and environmentally friendly compared to the previously mentioned processes.

In 2001, Saka and Kusdiana [30] conducted a research on biodiesel production from vegetable oils without any aid of catalysts. The oil-methanol mixture was heated above the supercritical temperature. Biodiesel was removed from the reaction mixture, and the excess methanol was removed by evaporation for 20 min at a temperature of 90°C. It was reported that 95% conversion was achieved in the first 4 min of reaction with optimum process parameters of alcohol : oil molar ratio of 42 : 1, pressure of 430 bar, and reaction temperature of 350°C. After one year (2002), Demirbaş [77] studied transesterification of six different vegetable oils in supercritical methanol and reported that increasing reaction temperature to supercritical condition had favorable influence on ester conversion.

Compared to catalytic reactions, SCM reactions are fast and can achieve high conversions in a very short time. However, the reaction requires higher temperatures, pressures, and alcohol to oil molar ratio in comparison to catalytic transesterification, which result in high production cost [67, 68].

It is clearly shown that the three transesterification processes presented have several drawbacks. They are energy intensive, recovery of byproduct is difficult, catalysts have to be removed, and waste treatment is required. To overcome these problems, enzymes have been proposed [7, 54, 79]. Most important advantage of using enzymes is their ability to convert FFA contained in the fat or oil to methyl esters completely. Additionally, glycerol, byproduct, can be easily recovered [26, 51, 80].

#### 2.1.3. Enzymatic Transesterification

There is a great interest on using biocatalysts to catalyze TG transformation to biodiesel, which has the advantage of having low operating conditions and high product purity. Enzymatic transesterification can be carried out at 35 to 45°C [41, 42, 81]. Contrary to chemical catalysts, enzymes do not form soaps and catalyze esterification of FFA and TG in one step without any need of the washing step. On the other hand, the major disadvantages of the enzymatic transesterification are its slower reaction rate and possible enzyme inactivation by methanol [27, 62, 82]. Lipase is an enzyme capable of catalyzing methanolysis reactions. It can be obtained from microorganisms such as bacteria and fungi. Lipases from *Mucor miehei*, *Rhizopus oryzae*, *Candida antarctica*, and *Pseudomonas cepacia* are the most commonly used enzymes [39, 62]. Lipases belong to a group of hydrolytic enzymes called hydrolases. In biological systems, lipases hydrolyze TGs to fatty acids and glycerol [66]. They work in mild conditions and have an ability to work with TGs from different origins. They have the ability to catalyze transesterification of both TGs and FFAs to give esters.

Extracellular and intracellular lipases are the major biocatalyst [5, 56]. Extracellular lipases refer to the recovered enzymes from the microorganism which is then purified, whereas intracellular lipases, the enzyme remains inside the producing cell walls [62]. In term of regioselectivity, lipases have been divided into three types [81]:

- sn-1,3-specific: hydrolyze ester bonds in positions *R_1_* or *R_3_* of TG,
- sn-2-specific: hydrolyze ester bond in position *R_2_* of TG,
- nonspecific: do not distinguish between positions of ester.

Fjerbaek et al. [39] stated that for biodiesel production from TG, lipases should be nonstereospecific where all TG, DG, and MG can be converted to fatty acids methyl esters (FAME). In addition, they should also be able to catalyze FFA esterification. 

Despite the lipases advantages over acid and base catalysts, lipases are costly which limit their industrial use [60, 83]. For that reason, reusability of the enzyme by using it in an immobilized form is essential from economic point of view.

Soluble enzyme acts as a solute in that they are dispersed in the solution and can move freely, but at the same time difficult to separate and to handle. One promising approach to overcome this difficulty is to immobilize the enzyme in a way that can be separated later by any simple separation method. Enzyme immobilization is a technique where free movement of the enzyme is restricted and localized to an inert support or carrier. This technique has many advantages, the most important of which is that the immobilized enzyme can be reused [40, 84]. In addition, by immobilization, the operating temperature of the process can be increased [39]. Cao [84] mentioned that an immobilized enzyme has to perform two essential functions, namely, the noncatalytic functions that are designed to aid separation and the catalytic functions that are designed to convert the targeting substrates within a desired time. This is in addition to the fact that the process is environmentally friendly and more sustainable [82]. 

**Figure 1:**
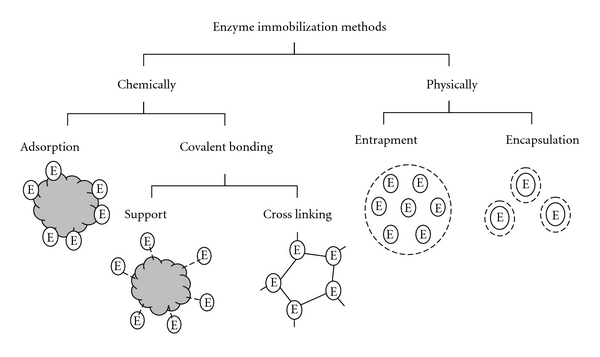
Enzyme immobilization methods.

Enzyme immobilization can be carried out in different ways. It can be classified into chemical and physical methods as shown in Figure 1. In biodiesel enzymatic production, various immobilization techniques have been used. Du et al. [24] used adsorption on macroporous resin, Noureddini et al. [27] worked on hydrophobic sol-gel support by entrapment, and Orçaire et al. [28] worked on silica aerogel by encapsulation. 

Amongst all possible immobilization methods, physical adsorption has been clearly selected by most researchers due to its ease, the absence of expensive and toxic chemicals, ability to retain the activity, and feasibility of regeneration [54]. But, immobilized enzymes are also subjected to diffusion limitation (internal and external) and inactivation (mostly by methanol) [85]. These problems have been studied and solved by different researchers.

To overcome immobilized lipase inactivation, the addition of an inert solvent has been suggested. However, solvent addition is not highly recommended since this will require using solvent recovery units, which will increase production cost. 

Köse and coworkers [86] investigated lipase-catalyzed transesterification of cotton seed oil with methanol in solvent-free medium. Yield of 92% was achieved in the presence of the Novozym 435 in 24 h reaction. This was performed at 50°C, 4 : 1 alcohol to oil and 30% enzyme loading. In 2007, Royon et al. [87] performed comparable work for cotton seed oil using Novozym 435 at the same condition, but with using *tert*-butanol as solvent. They noted that *tert*-butanol dissolved both methanol and glycerol that might inhibit enzyme activity, and a higher conversion of 97% was observed after 24 h of reaction. Similarly, Nelson et al. [33] tested the effect of using solvent on biodiesel production yield. By using *Mucor miehei* lipase, yield of 94% was obtained in *n*-hexane system, whereas only 19% yield obtained in a solvent-free system after 8 h reaction with methanol. On the other hand, ethanol produced 65% yield in solvent-free system and 98% in n-hexane system within first 5 h of the reaction. In the same approach, using 80% *tert*-butanol (based on oil weight) improved biodiesel yield from soybean oil deodorizer distillate with 4% Novozym 435 from 80 to 84%. However, further increase in solvent use decreased the yield, which might result from the dilution effect on reactants [88]. Wang et al. [88] obtained same yield, 84%, when a combination of 2% Novozym 435 and 3% Lipozyme TL were used. 

The importance of using solvents has been addressed well in the literature. However, as mentioned earlier solvents can be toxic, flammable, and have to be separated from the ester for reuse. Hence, efforts have been made to offer alternative solvents that are nontoxic and environmentally friendly. Candidate solvents that can replace previously mentioned solvents should have same advantages of dissolving both substrates and reduce excess alcohol inhibition and at the same time avoid the drawbacks of difficult separation of the solvent. In this regard, supercritical fluids (SCF) have been suggested as alternative solvents [89]. Further discussion on the use of SC-CO_2_ as a reaction medium is found in Section 4.1.2.

### 2.2. Biodiesel Feedstocks

Biodiesel can be synthesized from a great variety of feedstocks. These feedstocks include most vegetable oils (soybean oil, jatropha oil, rapeseed oil, palm oil, sunflower oil, corn oil, peanut oil, canola oil, and cottonseed oil) and animal fats (tallow and lard). They can also be produced from other sources like waste cooking oil, greases, and oleaginous microorganisms with excess microbial lipid such as microalgae [13].

#### 2.2.1. Vegetable Oils

Since vegetable oil is a feedstock that is available in large quantities, it has been widely used for the conversion to biodiesel. Majority of vegetable oils have been employed for biodiesel production such as soybean oil [44, 90], rapeseed oil [30, 80], canola oil [64], palm oil [45, 54, 91], and sunflower oil [92, 93]. However, producing biodiesel from vegetable oils competes with their use as food and involves additional land use. Also, in industrial scale, biodiesel production requires considerable use of arable lands.

#### 2.2.2. Waste Cooking Oils

Fried oils and fats are usually broken down after a period of use and become unsuitable for further cooking as a result of increasing of FFA content. Once this reached, they are discarded or recycled. This type of feedstocks is of low cost, making them attractive for fuel production [46]. Using waste cooking oil, especially those that cannot be treated, will reduce the environment pollution. Waste cooking oil conversion into biodiesel through the transesterification process reduces their molecular weight to approximately one-third, viscosity by about one-seventh, as well as reducing their flash point and volatility [4, 51]. High oil conversion (>90%) has been reported by many investigators [94–96] in spite of the high FFA contents that range from 5 to 15 wt%.

#### 2.2.3. Animal Fat

Animal fats are received from cattle, hog, chicken, lamb, and fish. Tallow and animal meats which are not allowed to be used as food can be used as biodiesel feedstock. However, these two sources have discontinuity problem in their supply. It is possible that suddenly a high bulk of material is available followed by a period with no supply like in the case of animal disease [48]. Animal fats are characterized by the high amount of saturated fatty acids (SFA). They are solid at room temperature and cannot be used as fuel in a diesel engine in their original form [97]. Authors of this paper had investigated possibility of biodiesel production from lamb meat fat [98] and tallow [99, 100] as feedstock.

#### 2.2.4. Oleaginous Microorganisms

As an alternative to vegetable oils and animal fats, oleaginous microorganisms have recently attracted great attention. It has been reported that such microorganisms accumulate oils and have microbial lipid content exceeding 20% [13]. The scope of this paper is on the use of microalgae in biodiesel production.

Using algae as a feedstock has been studied worldwide by several decades. However, for biodiesel production, this was started by an 18-year National Renewable Energy Laboratory (NREL) research project [50]. The potential of using algae for biodiesel production can be seen from their ability to produce large amount of biodiesel and reduce the production cost. Based on algae size, they are classified to macroalgae and microalgae. Macroalgae are large and multicellular, whereas microalgae are small and unicellular. Due to the simple cell structure, microalgae are widely used and have been accepted as promise feedstock. The following section gives more details about microalgae and their potential as feedstock for biodiesel production.

## 3. Microalgae

Algae that contain chlorophyll are photosynthetic microorganisms that convert inorganic carbon, such as carbon dioxide, in the presence of light, water and nutrients to algal biomass [101–106]. Majority of algae are living in aquatic (saline or freshwater) environments, whereas some of them can be found in other environments such as snow, desert soils, and hot springs [107]. They can be either autotrophic or heterotrophic. Autotrophic algae require only carbon dioxide, light, and salts to grow, whereas heterotrophic require an organic source of carbon, like glucose, as well as nutrients [105, 108–110]. However, heterotrophic algae are not as efficient as autotrophic algae for oil production [49, 111]. Autotrophic is more favorable as it does not require glucose which is a food source and at the same time fixes CO_2_, which has positive effect on the environment. Microalgae also can be either phototrophic or chemotrophic. Phototrophic algae use light as an energy source, whereas chemotrophic type use oxidizing compounds [107]. Additionally, some algae are capable of behaving in both autotrophic and heterotrophic modes. These are called mixotrophic algae [104, 112].

Algae range from unicellular to multicellular forms [105]. Some algae are motile while others are nonmotile. Moreover, they may exist as colonies, filaments, or amoeboids [104]. Based on their internal structure, algae cells are generally categorized into eukaryotes and prokaryotes. Prokaryotic cells do not have nuclear membrane-bound DNA, organelles and other membranous structures as eukaryotic cells. As shown in Table 1, almost all the algae are eukaryotes. In eukaryotes, microalgae cells consist of cell wall, plasma membrane, cytoplasm, nucleus, and organelles such as mitochondria, lysosomes, and golgi. 

As shown in Table 2, microalgae oil contents are usually between 20–50% of dry algae biomass weight. However, many microalgae oil content may exceed 80% of dry algae biomass weight [49, 101, 133, 134]. Besides, microalgae can grow very fast by doubling biomass in 24 hours, and during exponential growth phase they can double their biomass in about 3.5 hours [49, 111, 134, 135]. 

Algal species may change their composition, shape, and color based on growing culture and growth condition such as light, nutrients, temperature, and acidity, pH. It is well known that using stressful environment may cause algae to store more oil.

Unlike glycerolipids that are found in membranes under optimal conditions, many microalgae alter towards accumulations of neutral lipids in form TAG [136]. Microalgae composition is species specific and varies between different microalgae depending on nutrient, salinity, medium pH., temperature, light intensity, and growth phase. In all cells, lipid and fatty acids are constituents that act as membrane compounds, storage product, metabolites, and energy source. It is known that under stress condition, photosynthesis activity decreases; therefore, lipid synthesis occurs. Most of microalgae-produced oils having fatty acid constitutions similar to most common vegetable oils [137].

In general, lipids may include neutral lipids (nonpolar), polar lipids, wax esters, sterols, and hydrocarbons as well phenyl derivatives [138]. Major part of nonpolar lipids of microalgae is TGs and FFA. Typically, algae lipids have a carbon number range C_12_–C_22_. Most of fatty acids found in algae lipids are straight chain with even number of carbon atoms. They may be either saturated or unsaturated [115].  Table 3 gives a summary of the range of lipid reported in different algae species.

Microalgae are classified into four main classes according to their pigment components: diatoms (*Bacillariophyceae*), green algae (*Chlorophyceae*), blue-green algae (*Cyanophyceae*), and golden algae (*Chrysophyceae*) [102, 103, 108, 139].  Table 4 gives a brief description of each division.

Microalgae biomass contains three main components: protein, carbohydrates, and lipids and, therefore, can be used in different applications ranging from food products to biofuels. They are usually used as animal feed [140], human health food [104, 141], and as biofertilizer [49]. Additionally, microalgae can be used for atmospheric CO_2_ mitigation. It was reported that there are over 40,000 species of algae [136], but only limited number of these have been studied and have commercial significance [142].

### 3.1. Potential of Using Microalgae as Feedstock for Biodiesel Production

Biodiesel production from microalgae oil is more promising and sustainable alternative to previously mentioned feedstocks (Section 2.2) [143]. Compared to plants, algae do not compete with food crops and have higher energy yields per area than terrestrial crops. They present a good source of renewable biofuels, which include methane via anaerobic digestion of algal biomass, biodiesel derived from microalgae oil, and biohydrogen via photobioproduction [133, 144, 145]. The focus of this paper is on biodiesel.

Using microalgae as a fuel source is not new; it was suggested more than 60 years ago by Meier for methane production [136]. However, only recently microalgae received noticeable attention due to increasing environmental concerns. Main advantage of using microalgae as feedstock is their rapid growth potential with short biomass doubling time (3.5 hours) during exponential growth and oil content ranging from 20 to 50% dry weight of biomass for numerous microalgae species, as shown in Table 2. Other major advantages and features of using microalgae are the following.

- Ability to grow in nonarable land [49, 126, 144, 146, 147] where they can be cultivated on lands that is unsuitable for agriculture, that is, waste land [103, 148]. Therefore, biodiesel production would not be in any way competing with food production [106, 149].
- Can be cultivated in saline and brackish environments leading to reduction in fresh water load [111].
- Daily harvesting [49, 126, 150] and short harvesting cycle [106, 111] in comparison to crop plants.
- High photosynthetic efficiency due to their simple structure [111, 147, 148].
- Reduction in major greenhouse gas contributor, by utilizing CO_2_ from industrial flue gasses [49, 126, 146, 149–151]. Thereby, they are considered as CO_2_ fixers [152].

Table 5 compares different sources of biodiesel and their oil yield per area. Chisti [49] mentioned that in order to satisfy with the US demand for transportation fuel, the biodiesel industry would need to produce 530 billion liters annually. As can be found from Table 5, the most feasible biodiesel source for the US is microalgae.

Consequently, biodiesel production from microalgae is considered to be the best efficient feedstock for biodiesel production to displace conventional feedstock's and meet global demand of fuel [153].

To use microalgae for the production of biodiesel, several processes have to be carried out. These consist of strain selection, cultivation, harvesting, extraction of the oil, and production of biodiesel from extracted oil, in which each step can be accomplished with various technologies. These steps are detailed in the following sections.

### 3.2. Microalgae Oil Production Systems

#### 3.2.1. Microalgal Strain Selection

Microalgae come in a variety of strains; each has different proportions of lipid, protein, and carbohydrates contents. From over 3,000 collected, screened, and characterized algal strains in the National Renewable Energy laboratory (NREL) sponsored project [50], selection of the most suitable strain needs certain parameters evaluation. These parameters include oil content, growth rate and productivity, strain adaptableness, and withstanding to different weather conditions such as temperature, salinity and pH, and high CO_2_ sinking capacity and provide valuable coproducts [108]. Thus, right strain selection is critical.

Numerous researches have been carried out on different species tolerance. Many of them were found to be suitable for biodiesel production. As shown in Table 6, some microalgae have high lipids content such as *Nannochloropsis sp.*, and others have high protein contents like *C. protothecoides* (autotrophic) while others have high carbohydrates content like *Oscillatoria limnetica *under normal conditions. Among possible microalgae strains for biodiesel productions are *Chlorella* species. As can be seen from Table 2, *C. protothecoides *oil content is roughly 15% (% dry weight) under control conditions, but this can reach around 44% [154, 155], 53% [156], and 55% [120] when grown heterotrophically. 

On the other hand, generally, lower oil strains grow faster than high oil strains. This is due to slow reproduction rate as a result of storing energy as oil not as carbohydrates [157]. In addition, it should be taken into consideration that some microalgae contain high levels of unsaturated fatty acids, which reduce the oxidative stability of the biodiesel produced [158–160]. 

Rodolfi et al. [126] have screened variety of microalgal strains by evaluating biomass productivity and lipid content in 250-mL flask laboratory cultures (Table 7). Strains that have shown some promise lipid productivity can be further improved genetically. Genetic engineering can improve all aspects of algal production, harvesting, and processing for enhanced biodiesel capabilities.

#### 3.2.2. Microalgal Biomass Production

The main way to produce microalgal biomass is the cultivation. For commercial biomass production, microalgal biomass must be easily cultivated in the required scale. Microalgae cultivation can be carried out either via photoautotrophic methods in open systems (open-ponds) [153, 161] or closed systems (photobioreactors) [102, 161, 162], or via heterotrophic methods [120, 154]. All methods have their advantages and disadvantages; therefore, investigators disagree about which of the methods and systems is more favorable. Choosing best biomass production method or system depends on the selected algal strain and its integration with appropriate downstream processing which is the means for affordability and scalability of biodiesel production.

**Photoautotrophic**

As mentioned previously, photoautotrophic microalgae need light and carbon dioxide as energy and carbon sources, respectively. Thus, photoautotrophic algae cultivation is carried out in the presence of light in open ponds and photobioreactors.

Open ponds are the most commonly used systems, and their structure has been well documented. Open ponds are made of a closed loop with recirculation channels. A paddlewheel that continuously operates is usually used to prevent sedimentation and provide mixing. During daylight, the culture is fed continuously in front of the paddlewheel where the flow begins and circulates through the loop to the harvesting point. On completion of the circulation loop, broth is harvested behind the paddlewheel [49, 102, 161, 163]. Inclined, circular, and raceway ponds are operated at large scale. On the other hand, photobioreactors are closed bioreactors, which are designed as tubular, plate, or bubble column reactors. Among these, the most common type is tubular photobioreactors. These consist of less than 0.1 m diameter transparent tubes made from plastic or glass. Tube diameter is a critical design criteria as light does not penetrate too deeply in dense culture broths [49]. This leads to O_2_ accumulation and thus inhibits the photosynthesis process.

Typically, open ponds are the preferred large scale cultivation system [49]. This is due to their simplicity and low construction and capital costs [163]. However, these systems are open to the atmosphere, which lead to water evaporation and unwanted species contaminations. Besides, cell's poor utilization of light and low mass productivity, due to the low CO_2_ deficiencies and inefficient mixing, are other limitations [151, 164]. Therefore, for water, energy, and chemicals saving purposes, photobioreactors have been proposed, but they are not yet commercialized.

Main advantages of using photobioreactors are better algal culture and environment controlling [163], large surface to volume ratio, less water evaporation, better isolation from outside contaminations, and higher mass productivity [158]. However, photobioreactors are usually made of plastic, and UV deterioration of plastic surface is the main disadvantage. In addition, biofilm formation will require periodic cleaning [165].  Table 8 shows a comparison between the two photoautotrophic cultivation methods.

For a cost-effective cultivation, a combination of the two previous mentioned systems, referred to as hybrid system, is the most logical choice [153]. In this type of systems, microalgal strain with high oil content is grown in photobioreactors in nutrient and CO_2_-rich conditions firstly to promote rapid reproduction; then the microalgae enter an open system with limited nutrient to encourage oil production [166]. This process has been successfully verified by Huntley and Redalje [167].

In addition to microalgae strains influence on oil accumulation, cultivation parameters like temperature, light intensity, pH, water salinity, and nitrogen sources also influence oil production. It has been reported that the lipid content in various microalgae strains from *Chlorella* species increased when growing in low-nitrogen media compared to nitrogen-rich media [168, 169]. However, in these low-nitrogen media, a reduction in growth rate was reported. Similar results were also found by Widjaja et al. [170]. Since the cell needs sufficient nitrogen for growth, the cell production and division may reduce in the low-nitrogen media. However, carbon metabolism continues leading to utilize more energy for oil production rather than biomass growth [50, 171].

Other factors like CO_2_, light intensity, and temperature also significantly affect microalgae lipid content and composition. Renaud et al. [119] investigated the effect of temperature within the range of 25 to 35°C on *Rhodomonas* sp., *Chaetoceros* sp., *Cryptomonas* sp., and *Isochrysis* sp. growth rate and lipid content. Their results showed that optimum growth temperature was 25–27°C for *Rhodomonas* sp., and 27–30°C for *Cryptomonas* sp., *Chaetoceros* sp., and *Isochrysis* sp. Only *Chaetoceros* sp. was able to grow at 33 and 35°C.

With the intent of providing sufficient light to the cultivation systems, open ponds are usually made shallow, and tabular reactors are made small in diameters. Tang et al. [172] studied the influence of the above mentioned parameters on *Dunaliella tertiolecta *growth, lipid content, and fatty acid composition. It was reported that increasing light intensity increases cell growth rate regardless of the light source. On the other hand, as for the CO_2_ effect, the highest growth rate was found when CO_2 _ concentration was in the range of 2 to 6%.

**Heterotrophic Cultivation**

Unlike photoautotrophic microalgae, heterotrophic species are cultivated in a dark environment by utilizing organic carbon as carbon and energy sources [109, 173]. Heterotrophic cultivation method depends on the microalgae ability to eliminate light requirement and assimilate organic carbon [174]. This solve light limitation problem that appears with photoautotrophic cultivation methods. However, not all microalgae are able to assimilate the organic carbon. Thus, this cultivation method has been studied in a limited number of microalgae species [120, 154, 175]. It has been reported that heterotrophic cultivation provides high oil content and high biomass productivity [120, 121, 154, 176, 177]. Liu et al. [178] compared lipid content of *Chlorella zofingiensis* cultivated under heterotrophic and photoautotrophic conditions. Lipid content of 51 wt% and 26 wt% were obtained, respectively. Liang et al. [174] compared *C. vulgaris* cell growth rate and lipid productivity under autotrophic and heterotrophic conditions, evaluated glucose, acetate, and glycerol carbon sources uptakes.  Table 9 illustrates the results obtained.

#### 3.2.3. Harvesting Technologies

After algal cultivation, biomass needs to be separated from the culture medium using one or more solid-liquid separation steps. Due to the microalgae small size (3–30 *μ*m) [102, 179, 180] and cultures medium dilution (less than 1 g L^−1^), microalgae need to be concentrated to simplify the lipid extraction step. Biomass recovery is difficult [181] and require dewatering using suitable harvesting method [49, 151, 182]. 

Usually, microalgae are harvested by centrifugation, filtration, or sedimentation. Sometimes these require a pretreatment, flocculation step to improve recovery efficiency [183, 184].  Table 10 summarizes the advantages and disadvantages of each method. 

 To conserve energy and reduce costs, algae are often harvested in a two-step process. In the first, algae are concentrated by flocculation where diluted culture is concentrated to about 2–7% total suspended solids [108, 183]. In the second step, cells are further concentrated using conventional harvesting methods to get an algae paste of 15–25% total suspended solids [183]. Algae harvesting cost can be high due to their low mass fraction and algal cell negative charge [182]. It is reported that microalgal cell recovery accounts for at least 20–30% of total biomass production cost [184, 185]. Harvesting technique selection depends on microalgal cells size and density, biomass concentration, culture conditions, and value of target product [102, 186].

**Flocculation**

Flocculation is a process that collects dispersed cells into aggregate to form large particles that facilitate cell broth separation by addition of chemical additives (flocculants). It is considered as pretreatment stage preceding the main harvesting process [184]. The main problems facing the flocculation step are the high cost and toxicity of the flocculent [187].

To endorse flocculation, chemical additives that bind algae or affect interaction between algae have to be used. There are two main types of flocculants inorganic and organic polymer. A large number of chemicals have been tested as flocculants for microalgal flocculation where the most effective one was aluminum sulfate and certain cationic polymers [187]. Numerous reports have been published concerning the flocculation of algal biomass. Among them is the work of Tenney et al. [188] which looked into fresh water microalgae flocculation using organic polyelectrolytes where extent of microalgal flocculation was determined. Cationic polyelectrolyte polyamine was found to flocculate the algae successfully at an optimum dose of 2.5 mg/L.

**Filtration**

Filtration separation method makes use of a permeable medium that has an ability to retain the biomass and allows the liquid to pass through. Surface and depth filtration systems are the two known types of filtration. In surface filtration, solids are deposited on the filter medium whereas in the depth type solids are deposited within the filter medium [189]. This is satisfactory for recovering large microalgae and not for algae that size approach bacterial dimension.

**Sedimentation**

Sedimentation is a technique that separates microalgae biomass suspension into a concentrated slurry and clear liquid based on gravity action and particle diameter. If the biomass to be separated is small in size, settling rate will be low, and flocculants addition will be inevitable. This is a low cost process; however, its reliability is low.

**Centrifugation**

Almost all types of microalgae can be separated from the culture medium by centrifugation. A centrifuge is mainly a sedimentation tank with an enhanced gravitational force, by centrifugation, that increases the rate of sedimentation. Biomass recovery depends on biomass residence time within centrifugal field, settling rate, and distance [184]. Centrifugal recovery can be rapid, but it is energy intensive. Nevertheless, this process is a preferred method of recovering algal cells [183, 184]. Currently, there is no low cost harvesting method for all strains.  Table 10 summarizes advantages and disadvantages of each harvesting method.

#### 3.2.4. Drying

Following harvesting of the algal biomass, algal slurry moisture content has to be reduced to at least 10% by drying and dehydration. Numerous methods have been employed for drying. Most common methods are sun drying, spray drying, drum drying, and freeze drying. Again, best drying method selection depends on required operation scale and desired product value.

In biodiesel production, lipid-rich feedstock with low water content is required; therefore, microalgae drying has to be carried out. However, drying step is energy intensive, which adds to the cost complexity of the overall production process. Various drying systems differ in both energy and cost requirements.

Sun drying is an old and cheap drying method that can be performed easily by exposure to a solar radiation source. However, it takes long drying time, requires large drying surface area, and might result in loss of products. In addition, sun drying, unlike drum drying, does not have any sterilization effect of the dried sample. On the other hand, spray drying is a method that can be used for high value products, but it has the disadvantage of being expensive and might cause significant deterioration of algae [184]. In contrast, freeze drying has been commonly used by many investigators. Freeze drying has the advantage of breaking up species cells and turning them into fine powder that makes homogenization unnecessary [190]. Belarbi et al. [191] reported that freeze-dried sample can be subjected easily for oil extraction without cell disruption. Freeze driers have been used in algae lipid extraction to extract lipid from *I. galbana* [192], *P. tricornutum *[193], *C. vulgaris* [194], *S. platensis* [195], and *Chlorella sp*. [196].  Table 11 summarizes advantages and disadvantages of each technique. However, freeze drying is a slow process and requires very high capital investment.

#### 3.2.5. Oil Extraction

As stated previously, effective extraction requires concentrated algae solution. Thus, a high degree of algae concentration which takes place in the harvesting step is necessary before biomass lipid extraction. Typically, there are four well-known methods to extract oil from microalgae: (1) expeller/press, (2) solvent extraction with hexane, (3) subcritical water extraction, and (4) supercritical fluid extraction.

The recovering of intracellular products like oils from microalgae is usually difficult due to the cell wall robust structure [151, 197]. Therefore, prior to lipid extraction, algae cell has to be disrupted to a degree that facilitates extraction step [184, 198]. Several methods can be used to disrupt cell membrane. They include homogenizer, bed mill, ultrasound, autoclaving, freezing, and osmotic shock [151]. Among them, homogenizers and bed mills are often preferred because of their short residence time and lower operating costs [175]. Chisti and Moo-young [197] reviewed microbial cell disruption for intracellular products.

**Mechanical Extraction**

Mechanical oil extraction includes expeller press and ultrasonic extraction. In pressing technique, the presser crushes and pushes the oil out of the dry microalgal biomass. Despite expeller simplicity and lower investment cost, low oil recovery yield, high power consumption, and maintenance cost are the limitations [199].

In ultrasonic technique, shock waves break the walls and release oil to solvent. These waves are created when bubbles (created by ultrasonic wave associated from ultrasonic reactor) collapse near cell wall. Ultrasonic extraction has an advantage of being fast and efficient; at the same time it needs large amount of solvent, especially in case of low sample concentration. That's because at low concentrations, samples need to be extracted more than once using new fresh solvent [200].

**Chemical Extraction**

The well-known concept of “like dissolve like” is the basic of the Bligh and Dyer [201] solvent extraction method. This method is the most widely used for extracting lipids from microalgae, wherein hexane is one of the most widely used solvent due to its high extraction capability and low cost.

For successful lipid extraction using an organic solvent, the solvent must be able to penetrate through the matrix to contact and dissolve the lipid. When hexane is used as a solvent, it is mixed with the algal biomass and is then separated by filtration. The solvent has to be separated from the extracted oil using distillation which is energy intensive. Miao and Wu [120] reported that large amount of microalgal oil was efficiently extracted from *C. protothecoides* using *n*-hexane. Beside, cosolvent combinations have been used by many other investigators [192, 193, 202, 203]. Hexane/ethanol and hexane/isopropanol cosolvents have been commonly used in microalgal lipid extraction. The polar solvent, which is the alcohol, is first added to disrupt the algal cell membrane. This will enhance the ability of the hexane to extract almost all the lipids. The cosolvent is then removed by liquid-liquid extraction with water. The hexane solvent extraction method can also be used in combination with the oil press/expeller mechanical method. After extracting the oil from the algae using the expeller, the remaining pulp is then mixed with hexane in order to remove any remaining oil. In this combined method, more than 95% of the total oil present in the algae is extracted [204].

The selection of lipid extraction methods depends on the extraction efficiency. Therefore, a method of high performance, such as chemical extraction, is favored over the less efficient methods, such as mechanical extraction, despite the organic solvents negative environmental impacts.

To avoid the environmental impacts of using organic solvent, nontoxic solvents have been suggested, such as subcritical water (SCW) and SC-CO_2_. The SCW extraction operates at temperatures just below the critical temperature, 374°C, and at high pressure, usually from 10 to 60 bar, that maintains the water in liquid form. At these conditions, water becomes less polar, and lipids can be solubilized easily. Additionally, using water at subcritical condition can eliminate the dewatering step, and high-quality product within short extraction time can be achieved [205]. However, reaching the above mentioned temperature requires large energy consumption.

On the other hand, supercritical fluids extraction makes use of fluid's salvation power enhancement when reached above their critical point. Due to supercritical carbon dioxide's preferred critical properties, low toxicity, biodegradability, and availability, it has been used to extract many desired compounds from solid matrix. Other attractive point of using SC-CO_2_ as extraction solvent is that after extraction, solvent and product can easily be separated once the temperature and pressure are lowered to atmospheric conditions.

### 3.3. Microalgae Oil Production Costs

The idea of producing biodiesel from microalgae was the main focus of NREL project [50]. It is not much different from other biodiesel produced from vegetable oils, animal fats, or waste cooking oils. It was reported that biodiesel from vegetable oil and waste grease roughly costs $ 0.54 to $ 0.62/L and $ 0.34 to $ 0.42/L, respectively [206]. Chisti [49] reported that biodiesel from palm oil almost costs $ 0.66/L and in year 2006 petrodiesel average price was $ 0.49/L, which added about $ 0.14 to palm oil cost and 35% more than petrodiesel price. The objective is therefore to reduce the product cost to at least $ 0.48/L ignoring the effect of tax on biodiesel. However, the estimated cost of biodiesel increases to $ 0.72–1.4/L using microalgae with 70 wt% and 30 wt% (per dry-weight) oil content, respectively [49]. 

The high cost of biodiesel comes mainly from the high feedstock cost; 60–90% of biodiesel cost is estimated to be from the cost of the feedstock [7]. Therefore, looking for alternatives that are cheap became essential. From that point, microalgal oil production should be enhanced. Microalgae growth requires light, CO_2_, water, and salt utilization. To minimize production cost, oil production must rely on maximum available mentioned requirements. Therefore, using water from waste water treatment units that contain required growth nutrients and salts and diverting CO_2_ from power plants is desirable and beneficial. Other attributers are development of low harvesting process by genetic engineering, improvements in photobioreactors design, and coproduction of other high values products from residual biomass after lipid extraction [184].

## 4. Supercritical Fluids

**Figure 2:**
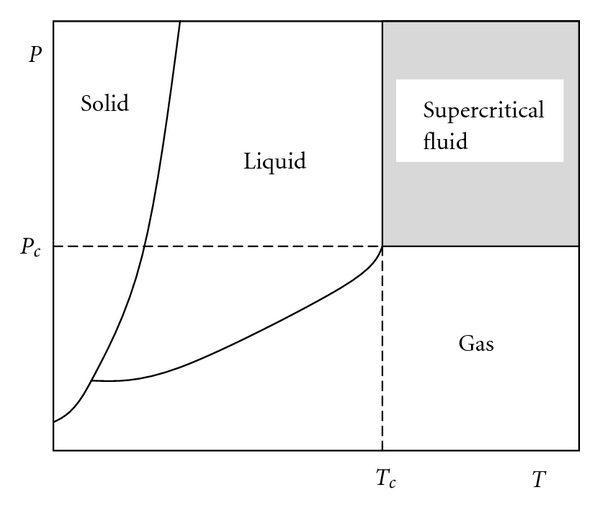
Pure component phase diagram.

Supercritical fluids (SCFs) are fluids at pressures and temperatures above their critical values. Critical values represent the highest temperature and pressure at which the substance exists as a vapor and liquid in equilibrium. This can be simply clarified from supercritical fluids phase diagram (Figure 2). 

As shown in Figure 2, there are three single phases, solid, liquid, and gas, where a substance may occur. If a mixture of two, or more, phases exists in these regions, a separation between the phases is distinct, as a result of the difference in properties of the different phases. In Figure 2, the solid curves between phases indicate the coexistence of two phases. On the other hand, at a point beyond the critical point neither fusion, as a result of pressure increase, nor vaporization, as a result of temperature increase, will take place, which was defined earlier as a supercritical region.  Table 12 presents the values of the critical temperatures and pressures of selected fluids most commonly used as extraction solvents [127].

In the SCF state, a solvent displays properties which are intermediate to those of liquid and gaseous states; SCFs have more desirable transport properties than liquids and better solvent properties than gases. The liquid-like density of a SCF gives high solvation power and facilitates solubility while the gas-like diffusivity gives excellent transport properties, which increases the rates of transfer from the substrate matrix to the SCF solvent as compared to that of liquid organic solvents [207]. Moreover, the low viscosity of SCFs which is close to that of the gases is an additional advantage. This last property gives rapid solvent penetration into a solid matrix [208].

### 4.1. Supercritical Carbon Dioxide as a Candidate Solvent

The ability of supercritical carbon dioxide, SC-CO_2_, to extract a solute depends on the compounds functional groups, molecular weight, and polarity. Near to its critical point, CO_2_ is a good solvent for nonpolar to slightly polar solutes with low molecular weight. It is an inert at most conditions, inexpensive, nontoxic, and environmentally friendly [43, 209]. Moreover, when using SC-CO_2_ as a solvent, no solvent residue remains in the extract since CO_2_ is in a gas phase at the ambient conditions. The critical temperature and critical pressure of CO_2_ are 31.1°C and 72.8 bar, respectively, which are not extremely high. SC-CO_2_ has been identified as a good alternative solvent for a number of applications including separation and reaction.

#### 4.1.1. SC-CO_2_: Extraction Solvent

Extraction is the process of removal of a solute from a matrix using a solvent which is able to dissolve the desired solute. This involves contacting the matrix with the solvent either in a single stage or in multiple stages for certain period of time and then separating the solvent. During extraction period, the solute transfers from the matrix to the solvent. Required time to achieve successful extraction depends on the solubility. That depends on extraction temperature, contact area between the solute and solvent, solvent viscosity, and solvent flow rate.

Other conventional solvent extraction techniques suffer from several drawbacks such as long extraction time and high solvent consumption, in addition to being labor intensive, difficult to automate, and often require a postextraction cleanup [210]. With these drawbacks, supercritical fluid extraction, SFE, has been proposed using the extraction solvent in its supercritical state.

SCFs were first observed more than a century ago in 1822. However, it has been developed as a novel separation technique only in the past two decades. From an economical point of view and in order not to thermally alter the properties of the extracted materials, SCFs are mostly used as in the approximate range of temperature up to 1.2 times the critical temperature, *T*_c_, and pressure up to 3.5 times the critical pressure *P*_c_ [211].

Although a number of substances could serve as solvents, CO_2_ is the most common. SC-CO_2_ has many applications, especially in food processing, which include decaffeination of coffee and tea, production of hops extracts, flavors extract from herbs, and extraction of edible oils. Friedrich and Pryde [212] extracted oil from soybeans using SC-CO_2 _ and achieved a yield almost to that using *n*-hexane. In order to extract polar compounds from a matrix, polar supercritical fluid should be used. Thus, using a nonpolar solvent, CO_2_ sometimes faces difficulties to extract certain compounds from a sample matrix. To overcome this limitation, modifier fluids can be used to increase extraction efficiency. Among all modifiers tested, methanol was the most commonly used by investigators such as Tonthubthimthong et al. [213] who extracted nimbin from neem seeds. Brewer et al. [214] who extracted cocaine from human hair and Aghel et al. [215] who extracted pennyroyal essential oil using SC-CO_2_.

Due to the attractive features of SC-CO_2_, it has been used and assessed to extract lipids from different strains of microalgae [128–132, 216]. Maximum yields of 13, 9, 25, 8, 6, and 3% have been reported from *C. vulgaris* [128, 132], *C. cohnii *[216], *Nannochloropsis *sp. [129], *S. platensis *[130], *chlorococum* sp. [131], and *S. maxima* [132], respectively. The lipids were extracted from dried biomass in a temperature range of 40°C–80°C and pressure range of 100–550 bars. The lower extract yield was due to the low lipid content of grown biomass. 

The extraction efficiency of SC-CO_2_ was compared to conventional solvent extraction methods.  Table 13 shows the extraction yields of lipids, defined as amount of extracted lipids per dry biomass weight, extracted from different strains of microalgae using SC-CO_2_, as compared to that of conventional solvent extraction. As shown, similar yields were reported when using SC-CO_2_ and *n*-hexane for extracting lipids from *S. platensis* [130] and *S. maxima* [132]. However, 25–40% lower yields were reported when comparing SC-CO_2_ to *n*-hexane and acetone extractions, from *C. vulgaris* [128]. A lower yield was also reported when comparing SC-CO_2_ and ethanol extractions from *S. maxima* [132]. However, other studies showed a better performance than *n*-hexane from *chlorococum* sp. and *Nannochloropsis *sp. [129, 131]. 

To further enhance the SC-CO_2_ extraction yields, the use of a cosolvent has been suggested. SC-CO_2_ with 10% ethanol as a cosolvent for lipid extraction from *S. maxima* has been reported [217]. By doing so, the extraction yield increased by 24% from 32% to reach 40%. This enhancement was explained by ethanol destruct effect on microalgal cellular walls.

#### 4.1.2. SC-CO_2_: Reaction Media

Majority of chemical processes are carried out in organic solvents, which in most cases are toxic and flammable. Furthermore, these organic solvents need to be separated from the desired product and recycled back. To avoid these drawbacks, SCFs are suggested as an alternative.

As mentioned earlier, SCFs have gas-like diffusivities and low viscosities, which reduce mass resistance between reaction mixture and the catalyst and therefore result in an increase of reaction rate. Among possible solvents that can be used in supercritical conditions to conduct transesterification reactions, CO_2_ was chosen due to its low critical temperature which make the process less energy intensive and more importantly below the denaturation temperature of the biocatalyst.

Kumar et al. [41] esterified palmitic acid with ethanol in temperature range of 35 to 70°C in the presence of three different lipases in SC-CO_2_. Their results showed that Novozym 435 was the best catalyst. In SC-CO_2_, Lipolase 100T and hog pancreas lipase showed similar results. Yields of 74, 44, and 40% were reached using Novozym 435, Lipolase 100T, and hog pancreas lipase, respectively, which were comparable to yield in solvent free system. Romero et al. [89] esterified isoamyl alcohol in SC-CO_2_ and *n*-hexane. They noted that similar esterification degree was obtained in both SC-CO_2_ and *n*-hexane systems; however, initial reaction rate was higher in SC-CO_2_. Laudani et al. [218] compared FFA esterification with 1-octanol over immobilized lipase from *R. miehei *(Lipozyme RM IM) using three different reaction media: SC-CO_2_, *n*-hexane, and solvent free systems. SC-CO_2_ showed the highest conversion followed by *n*-hexane then solvent-free system.

Although SC-CO_2_ has been used as a reaction media for enzyme esterification of FFA, limited work has been done on transesterification. D. Oliveira and J. V. Oliveira [219] compared enzymatic alcoholysis of palm kernel oil using *n*-hexane and SC-CO_2 _ systems. In SC-CO_2_, highest conversion of 63% was obtained using Novozym 435 as catalyst whereas in *n*-hexane Lipozyme IM provided the highest conversion of 77%. Rathore and Madras [61] produced biodiesel from Jatropha oil with Novozym 435 in SC-CO_2_. Optimum conditions were found to be 45°C, alcohol : oil molar ratio of 5 : 1, 30% enzyme loading, and 8 h with conversions of 60–70%. Varma and Madras [220] produced biodiesel from caster and linseed oils with Novozym 435 in SC-CO_2_, and 45% yield in methanol and 35% in ethanol were obtained from linseed oil, whereas a very low yield of less than 10% was obtained from castor oil. Varma et al. [221] synthesized biodiesel from mustard and sesame oils using different acyl acceptors at 50°C for 24 h reaction. Their results showed that using mustard oil, conversion of roughly 70% and 65% can be obtained using methanol and ethanol, respectively. On the other hand, using sesame oil, a conversion of round 55% was obtained from ethanol, whereas only 45% conversion was obtained with methanol.

Despite the advantages of using SC-CO_2_ as a reaction media for the enzymatic production of biodiesel, it has not been reported in any previous work on microalgae oil.

## 5. Microalgae for Biodiesel Enzymatic Production Using SC-CO_2_

### 5.1. Microalgae as a Feedstock in Conventional Process

Recently, feasibility of using microalgae to produce biodiesel, as an alternative to fossil fuels, received significant attention since they are rich in lipids. Species like *C. vulgaris*, *C. emersonii*, *Nannochloropsis* sp., *P. tricornutum*, and *T. suecica* have been reported in the literature for biodiesel production, where most of them were cultivated using glucose as a carbon source. However, glucose can be fermented directly to produce bioethanol.

Conventionally, microalgae have been used for biodiesel production using chemical catalytic reactions. Miao and Wu [120] studied biodiesel production from heterotrophic cultivated microalgae oil from *C. protothecoides* by 100% H_2_SO_4_ (based on oil weight) acidic transesterification. Biodiesel optimum conversion yield of 63% was obtained with 56 : 1 methanol : oil molar ratio at 30°C in 4 h reaction time. To overcome disadvantages of homogeneous catalysts, Carrero et al. [222] tested the ability of using hierarchical zeolites as heterogeneous catalyst.

With the target to reduce biodiesel production cost associated with oil extraction cost, *in-situ* transesterification, which is a direct conversion without solvent extraction, of the biomass oil to biodiesel has been performed [223, 224]. A conversion of 91% was achieved after 8 h of reaction at 60°C from Chlorella sp. [223] and 39, 40, 77, 78, and 82% were obtained from *Synechocystis* sp. PCC 6803 *Synechococcus elongates*, *Chlorella sorokiniana*, *T. suecica*, and *Chaetoceros gracilis* at 80°C [224]. The high conversion obtained with *Chlorella *species could be due to the use of a stirring reactor that enhanced the mixing and reduced mass transfer resistances.

### 5.2. Enzymatic Biodiesel Production from Microalgae

Similar to conventional feedstocks conversion, microalgae oil can also be converted to biodiesel using lipase. In this perspective, *C. protothecoides* is the only species that has been tested so far. Xiong et al. [225] produced biodiesel with 98% conversion from *C. protothecoides* with 30 wt% of *Candida *sp. lipase. Reaction conditions were 3 : 1 methanol : oil molar ratio, 10% water content, 38°C, pH of 7, and 12 h reaction. Similar conversion was obtained by Li et al. [154] at similar conditions but using 75 wt% of the immobilized lipase rather than 30 wt%. *C. protothecoides* was cultivated heterotrophically in both studies using glucose.

### 5.3. Enzymatic Production with SC-CO_2_ Reaction Medium

To overcome the lipase inhibition limitations, mainly by methanol and glycerol, chemical solvents that can dissolve both methanol and glycerol have the advantage of increasing conversion yield. However, the use of organic solvents is not recommended due to its harmful environmental input and the solvent extraction unit.

Using SC-CO_2 _ as a reaction media adds to the advantages of organic solvents in saving downstream processing cost where product purification is not necessary. Since solubility is greatly influenced by fluid temperature and pressure adjustments, separation can be easily achieved by a pressure reduction where the product and enzyme do not dissolve at room temperature.

Due to its advantages over conventional organic solvents, the application of the high cost SC-CO_2_ process may be justified in oil extraction from microalgae. However, its justification for biodiesel production may not be evident, despite its positive effect on reducing inhibition effects and easy product separation. Nevertheless, a combined continuous process of extracting oil from microalgae using SC-CO_2_ and the use of the extracted oil for biodiesel production using immobilized lipase in SC-CO_2_ in a one integrated system would economically be feasible. In this continuous process, the oil that is extracted from microalgae is already dissolved in SC-CO_2_ and can be fed directly to the enzymatic bioreactor to produce biodiesel without the need for further expensive pumping. In this way, the attractive advantages of performing the reaction in SC-CO_2_ media will be gained, avoiding at the same time the disadvantage of high pumping cost. Besides, using high pressure CO_2_ might not have significant negative effect of lipase stability. Lanza et al. [226] investigated the influence of SC-CO_2_ pressure on lipase activity and reported that the residual activity of Novozym-435 was approximately 90%. Previous study of D. Oliveira and J. V. Oliveira [219] on converting palm kernel oil to biodiesel using Novozym 435 showed that the rise in pressure in the range 60–90 bar actually results in an enhancement of initial reaction rate and conversion. However, at pressures beyond but 200 bar, a change in lipase structure may occur, which has a negative effect on the reaction. Therefore, the application of SC-CO_2_ in the enzymatic reaction system should not exceed 200 bar.

## 6. Conclusions

As verified in this paper, biodiesel produced from microalgae can realistically satisfy the global demand of diesel-fuel requirements. However, for cost effective production, this will not be applicable without microalgae biology and production processes enhancements. The potential of microalgal biodiesel production depends on selected microalgal strain and its ability to live in saline or use wastewaters and utilize CO_2_ as a sole carbon source. In addition, biomass recovery that usually requires high energy and oil extraction has to be optimized for effective low cost overall process production. Another important point to be taken into consideration is the ability to use spent biomass, after oil extraction, for the production of other valuable coproducts such as animal feed or fertilizers. 

The paper presents SC-CO_2_ as a promising oil extraction technique from microalgae, and lipase as a biocatalyst for biodiesel production instead of the conventional chemical catalysts that require feed purification. The use of SC-CO_2_ as a reaction media for the enzymatic production of biodiesel has also been discussed in the paper. Authors of this review, suggest future work to be done on designing an integrated SC-CO_2_ extraction/reaction process, whereby a stream of extracted oil-rich SC-CO_2_ from selected microalgae species is fed to a bioreactor containing lipase for enzymatic conversion of the oil into biodiesel.

## Figures and Tables

**Table 1:** Summary of different algal groups classification for different habitat types [113].

Kingdom	Division	Habitat			
		Marine	Freshwater	Terrestrial	Symbiotic
Prokaryota	Cyanophyta	Yes	yes	Yes	yes
	Prochlorophyta	Yes	yes	Not detected	yes

Eukaryota	Glaucophyta	Not detected	yes	Yes	yes
	Rhodophyta	yes	yes	Yes	yes
	Heterokontophyta	yes	yes	Yes	yes
	Haptophyta	yes	yes	Yes	yes
	Cryptophyta	yes	yes	Not detected	yes
	Chlorarachniophyta	yes	Not detected	Not detected	yes
	Dinophyta	yes	yes	Not detected	yes
	Euglenophyta	yes	yes	Yes	yes
	Chlorophyta	yes	yes	Yes	yes

**Table 2:** Oil content of some common microalgae [49, 114].

Microalgae	Oil content (% dry biomass weight)
*Botryococcus braunii*	25–80
*Chlorella protothecoides*	23–30
*Chlorella vulgaris*	14–40
*Crypthecodinium cohnii*	20
*Cylindrotheca *sp.	16–37
*Dunaliella salina*	14–20
*Neochloris oleoabundans*	35–65
*Nitzschia *sp.	45–47
*Phaeodactylum tricornutum*	20–30
*Schizochytrium *sp.	50–77
*Spirulina maxima*	4–9
*Tetraselmis suecia*	15–23

**Table 3:** Fatty acid composition of lipids of different microalgae [115].

Fatty acid	*Spirulina platensis*	*S. maxima*	*Scenedesmus obliquus*	*C. vulgaris*	*Dunaliella bardawil*
C_12 : 0_	0.04	traces	0.3	—	—
C_14 : 0_	0.7	0.3	0.6	0.9	—
C_14 : 1_	0.2	0.1	0.1	2	—
C_15 : 0_	traces	traces	—	1.6	—
C_16 : 0_	45.5	45.1	16.0	20.4	41.7
C_16 : 1_	9.6	6.8	8.0	5.8	7.3
C_16 : 2_	1.2	traces	1.0	1.7	—
C_16 : 4_	—	—	26.0	—	3.7
C_17 : 0_	0.3	0.2	—	2.5	—
C_18 : 0_	1.3	1.4	0.3	15.3	2.9
C_18 : 1_	3.8	1.9	8.0	6.6	8.8
C_18 : 2_	14.5	14.6	6.0	1.5	15.1
*α*-C_18 : 3_	0.3	0.3	28.0	—	20.5
*γ*-C_18 : 3_	21.1	20.3	—	—	—
C_20 : 2_	—	—	—	1.5	—
C_20 : 3_	0.4	0.8	—	20.8	—
Others	—	—	2.5	19.6	—

**Table 4:** Summary of different microalgae divisions [104, 113, 116].

Division	Examples	Occurrence	Photosynthesis pigments	Reproduction
Diatoms	*Coscinodiscus granii *			
	*Tabellaria*			
	*Amphipleura*	(i) Oceans	(i) Chlorophylls *a* and* c * (ii) *B-*carotene	(i) Vegetative (binary fission or fragmentation)
	*Thalassiosira baltica*	(ii) Freshwater		(ii) Asexual (akinete, exospores, endospores or homospores)(iii) Sexual (isogamous, anisogamous or oogamous)
	*Skeletonoma*	(iii) Brackish water		
	*Chaetoceros*			
	*Cyclotella*			
	*Chlorella sp.*			

Green algae	*C. vulgaris *			
	*C. protothecoides*			
		(i) Oceans		
	*S. obliquus*	(ii) Freshwaters	(i) Chlorophylls *a* and* b *	(i) Vegetative (binary fission or fragmentation)
	*Haematococcus pluvialis*	(iii) Moist		(ii) Asexual (akinete or exospores or endospores or homospores)
	*Nannochloris*	(iv) Terrestrial habitats.		(iii) Sexual (isogamous, anisogamous or oogamous)
	*D. salina*	
	*B. braunii*	

			(i) Divided in to two groups	
Blue-green algae	*S. platensis *	(i) Freshwater	(ii) Most species have chlorophyll *a* as only form of chlorophyll and phycobilins as pigments	(i) Vegetative (binary fission and fragmentation)
	*Synechococcus*	(ii) Marine		(ii) Asexual (akinete or exospores or endospores and homospores)
	*Cyanidium*	(iii) Terrestrial		
	*Oscillatoria*	(iv) Symbiotic		
	*Anabaena cylindrical*	(v) Associations	(iii) Some have two forms of chlorophyll *a* and *b* and lack phycobilins	

Golden algae	*Isochrysis* * galbana *	(i) Fresh water	(i) Chlorophylls *a* and *b *	(i) Asexual (zoospores or aplanospore, hypnospores)
	*Dinobryon balticum*	(ii) Marine	(ii) Some have chlorophylls *e* carotene *a* and *c *	(ii) Sexual (isogamous or anisogamous or oogamous)
	*Uroglena americana*	(iii) Terrestrial		

**Table 5:** Comparison between different biodiesel sources [49].

Crop	Oil yield (L/ha)	Land area needed (M ha)^a^	Percent of existing US cropping area^a^
Corn	172	1540	846
Soybean	446	594	326
Canola	1190	223	122
Jatropha	1892	140	77
Coconut	2689	99	54
Oil palm	5950	45	24
Microalgae^b^	136,900	2	1.1
Microalgae^c^	58,700	4.5	2.2

^a^For meeting 50% of all transport fuel needs of the United States.

^b^70% oil by wt in biomass.

^c^30% oil by wt in biomass.

**Table 6:** Chemical composition of various microalgae (% dry weight).

Microalgae	Carbohydrates	Protein	Lipids	Reference (s)
*Chaetoceros muelleri*	19.3	46.9	33.2	[117]
*I. galbana*	26.8	47.9	14.5	[118]
*Chaetoceros calcitrans*	27.4	36.4	15.5	[118]
*Isochrysis sp.*	12.9	50.8	20.7	[119]
*Prymnesiophyte *(NT19)	8.4	41.3	14.7	[119]
*Rhodomonas *(NT15)	6.0	57.2	12	[119]
*Cryptomonas *(CRF101)	4.4	44.2	21.4	[119]
*Chaetoceros *(CS256)	13.1	57.3	16.8	[119]
*C. protothecoides* ^ a^	10.6	52.6	14.6	[120–122]
*C. protothecoides* ^ b^	15.4	10.3	55.2	[120, 121]
*Microcystis aeruginosa*	11.6	30.8	12.5	[122]
*Nannochloropsis sp*	29.0	10.7	60.7	[123]
*S. obliquus*	15	50.0	9.0	[124]
*Oscillatoria limnetica*	50	44.0	5.0	[124]
*B. braunii*	18.9	17.8	61.4	[125]
*Botryococcus protuberans*	16.8	14.2	52.2	[125]

^a^Autotrophic cultivation.

^b^Heterotrophic cultivation.

**Table 7:** Biomass productivity, lipid content, and lipid productivity of 30 microalgal strains cultivated in 250-mL flasks [126].

Algal Group	Microalgae strain	Habitat	Biomass productivity (g l^−1^ d^−1^)	Lipid content (%)	Lipid productivity (mg l^−1^ d^−1^)
Diatoms	*Chaetoceros muelleri* F&M-M43	Marine	0.07	33.6	21.8
	*C. calcitrans* CS 178	Marine	0.04	39.8	17.6
	*P. tricornutum* F&M-M 40	Marine	0.24	18.7	44.8
	*Skeletonoma costatum* CS 181	Marine	0.08	21.0	17.4
	*Skeletonoma* sp. CS 252	Marine	0.09	31.8	27.3
	*Thalassiosira pseudonana *CS 173	Marine	0.08	20.6	17.4
	*Chlorella* sp. F&M-M48	Freshwater	0.23	18.7	42.1
	*Chlorella sorokiniana* IAM-212	Freshwater	0.23	19.3	44.7
	*C. vulgaris* CCAP 211/11b	Freshwater	0.17	19.2	32.6
	*C. vulgaris* F&M-M49	Freshwater	0.20	18.4	36.9

Green algae	*Chlorococcum* sp. UMACC 112	Freshwater	0.28	19.3	53.7
	*Scenedesmus* *quadricauda *	Freshwater	0.19	18.4	35.1
	*Scenedesmus* F&M-M19	Freshwater	0.21	19.6	40.8
	*Scenedesmus* sp. DM	Freshwater	0.26	21.1	53.9
	*Tetraselmis. suecica* F&M-M33	Marine	0.32	8.5	27.0
	*Tetraselmis* sp. F&M-M34	Marine	0.30	14.7	43.4
	*T. suecica* F&M-M35	Marine	0.28	12.9	36.4
	*Ellipsoidion* sp. F&M-M31	Marine	0.17	27.4	47.3
	*Monodus subterraneus* UTEX 151	Freshwater	0.19	16.1	30.4
	*Nannochloropsis* sp. CS 246	Marine	0.17	29.2	49.7

Eustigmatophytes	*Nannochloropsis* sp. F&M-M26	Marine	0.21	29.6	61.0
	*Nannochloropsis* sp. F&M-M27	Marine	0.20	24.4	48.2
	*Nannochloropsis* sp. F&M-M24	Marine	0.18	30.9	54.8
	*Nannochloropsis* sp. F&M-M29	Marine	0.17	21.6	37.6
	*Nannochloropsis* sp. F&M-M28	Marine	0.17	35.7	60.9
	*Isochrysis* sp. (T-ISO) CS 177	Marine	0.17	22.4	37.7
	*Isochrysis* sp. F&M-M37	Marine	0.14	27.4	37.8

Prymnesiophytes	*Pavlova salina* CS 49	Marine	0.16	30.9	49.4
	*Pavlova lutheri* CS 182	Marine	0.14	35.5	50.2

Red algae	*Porphyridium* *cruentum *	Marine	0.37	9.5	34.8

**Table 8:** Comparison between open ponds and photobioreactors.

Method	Advantages	Limitations
Open ponds	(i) Simple (ii) Cheap (iii) Easy to operate and maintain (iv) Low capital cost	(i) Poor light utilization (ii) Difficulties in light and temperature controlling (iii) Water evaporation (iv) Foreign species contaminations (v) Lower mass productivity

Photobioreactors	(i) High surface to volume ratio (ii) Higher mass productivity (iii) Less contaminations (iv) Less water losses (v) Better light utilization	(i) Scalability problem (ii) Costly (iii) Complex (iv) Cells damage cases (v) Biofilm formation

**Table 9:** Lipid content, biomass, and lipid productivities of *C. vulgaris* grown autotrophically and heterotrophically on different carbon sources.

	Heterotrophic cultivation			Autotrophic cultivation
	Acetate	Glucose	Glycerol	
Biomass productivity (mg l^−1^ d^−1^)	87	151	102	10
Lipid content (%)	31	23	22	38
Lipid productivity (mg L^−1^ d^−1^)	27	35	22	4

**Table 10:** Advantages and disadvantages of different microalgal harvesting methods.

Method	Advantage	Disadvantage
Flocculation	(i) High recovery yield (up to 22 TTS)	(i) Flocculants may be expensive
	(ii) Low energy requirement	(ii) Contamination issue may occur
		(iii) Marine environment high salinity may inhibit the process
		(iv) Long process period

Centrifugation	(i) Reliable	(i) Energy intensive
	(ii) Corrosion resistance	(ii) Expensive
	(iii) Easy cleaning	(iii) High speed may deteriorate the cell
	(iv) Rapid	(iv) Cannot be used for species <30 *μ*m

Filtration	(i) Reliable	(i) Filters may need to be replaced periodically
	(ii) Able to collect species of low density	(ii) Membrane blockage
		(iii) High maintenance cost
		(iv) May be slow
		(v) Head loss

**Table 11:** Comparison between common four microalgae drying methods.

Method	Advantages	Disadvantage
Sun drying	(i) Cheap (no running cost, low capital cost)	(i) Difficult (ii) Slow (iii) Weather dependent (iv) Require large surface (v) Contamination

Spray drying	(i) Fast (ii) Continuous (iii) Efficient	(i) Cost intensive (ii) Species deterioration (i.e. pigments)

Drum drying	(i) Fast (ii) Efficient (iii) Sterilization advantage	(i) Cost intensive

Freeze drying	(i) Gentle	(i) Slow process (ii) Cost intensive

**Table 12:** Critical properties of common solvents [127].

Fluid	Critical temperature (°C)	Critical pressure (bar)
Xenon	16.7	59.2
Carbon dioxide	31.1	72.8
Ethane	32.4	49.5
Nitrous oxide	36.6	73.4
Chlorodifluoromethane	96.3	50.3
Ammonia	132.4	115.0
Methanol	240.1	82.0
Water	374.4	224.1

**Table 13:** Comparison of SC-CO_2_ performance and other conventional extraction solvents on lipids extraction yields from microalgae biomass.

Microalgae species	SC-CO_2_	Other conventional solvents			Reference
		Acetone	Ethanol	*n*-Hexane	
*C. vulgaris*	13.3	16.8	—	18.5	[128]
*Nannochloropsis *sp	25	—	—	23	[129]
*S. platensis*	7.8	—	—	7.7	[130]
*chlorococum* sp	5.8	—	—	3.2	[131]
*S. maxima*	2.5	4.7	5.7	2.6	[132]
